# Risk–Benefit assessment of foods: Development of a methodological framework for the harmonized selection of nutritional, microbiological, and toxicological components

**DOI:** 10.3389/fnut.2022.951369

**Published:** 2022-10-20

**Authors:** Géraldine Boué, Ermolaos Ververis, Aikaterini Niforou, Michel Federighi, Sara M. Pires, Morten Poulsen, Sofie T. Thomsen, Androniki Naska

**Affiliations:** ^1^Oniris, INRAE, SECALIM, Nantes, France; ^2^Department of Hygiene, Epidemiology and Medical Statistics, School of Medicine, National and Kapodistrian University of Athens (NKUA), Athens, Greece; ^3^European Food Safety Authority, Parma, Italy; ^4^ENVA, ANSES, LSA, Maisons-Alfort, France; ^5^Risk Benefit Research Group, National Food Institute/DTU, Lyngby, Denmark

**Keywords:** RBA, nutrition, food safety, alternative proteins, edible insects, risk assessment, risk ranking

## Abstract

Investigating the impact of diet on public health using risk–benefit assessment (RBA) methods that simultaneously consider both beneficial and adverse health outcomes could be useful for shaping dietary policies and guidelines. In the field of food safety and nutrition, RBA is a relatively new approach facing methodological challenges and being subject to further developments. One of the methodological aspects calling for improvement is the selection of components to be considered in the assessment, currently based mainly on non-harmonized unstandardized experts’ judgment. Our aim was to develop a harmonized, transparent, and documented methodological framework for selecting nutritional, microbiological, and toxicological RBA components. The approach was developed under the Novel foods as red meat replacers—an insight using Risk-Benefit Assessment methods (NovRBA) case study, which attempted to estimate the overall health impact of replacing red meat with an edible insect species, *Acheta domesticus*. Starting from the compositional profiles of both food items, we created a “long list” of food components. By subsequently applying a series of predefined criteria, we proceeded from the “long” to the “short list.” These criteria were established based on the occurrence and severity of health outcomes related to these components. For nutrition and microbiology, the occurrence of health outcomes was evaluated considering the presence of a component in the raw material, as well as the effect of processing on the respective component. Regarding toxicology, the presence and exposure relative to reference doses and the contribution to total exposure were considered. Severity was graded with the potential contribution to the background diet alongside bioavailability aspects (nutrition), the disability-adjusted life years per case of illness of each hazard (microbiology), and disease incidence in the population, potential fatality, and lifelong disability (toxicology). To develop the “final list” of components, the “short list” was refined by considering the availability and quality of data for a feasible inclusion in the RBA model. The methodology developed can be broadly used in food RBA, to guide and reinforce a harmonized selection of nutritional, microbiological, and toxicological components and will contribute to facilitating RBA implementation, enabling the generation of transparent, robust, and comparable outcomes.

## Introduction

Safe and nutritious food is essential for humans. The increasing knowledge on health outcomes associated with food consumption has led to the emergence of risk–benefit assessment (RBA), a new decision support methodological framework, in the beginning of the 21st century. RBA aims to assess simultaneously potentially adverse and beneficial health outcomes linked to the exposure of humans to specific dietary components or foods (1, 2).

**FIGURE 1:**
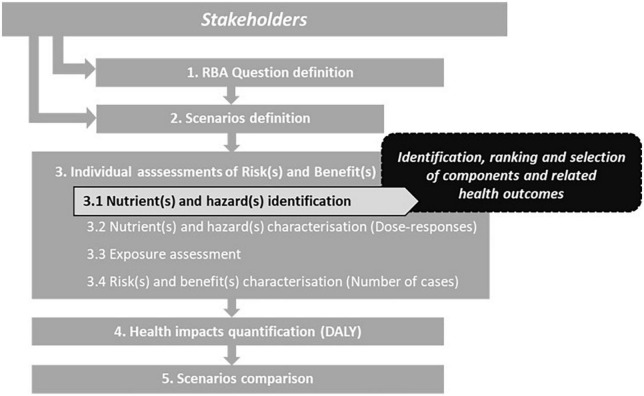
Risk–benefit assessment (RBA) stepwise approach [adapted from Assunção et al. (1) and Boué (42)].

The RBA methodology was developed from the traditional risk assessment paradigm (3), interconnected to risk–benefit management and risk–benefit communication. The RBA methodology (Figure 1) was initiated by EFSA (3, 4) and advanced through several European projects (1, 2, 5–9). RBA follows four steps of risk assessment, namely, hazard identification, hazard characterization, exposure assessment, and risk characterization, using an adapted strategy to enable the integration of both adverse and beneficial health outcomes linked to nutrition, microbiology, and toxicology.

Before conducting an RBA, it is necessary to define, through a close and continuous interaction between assessors and stakeholders the risk–benefit question and the respective exposure scenarios, to ensure a fit-for-purpose assessment for the targeted population groups. The reference (or baseline) scenario often corresponds to the current or zero exposure to a dietary element, and the alternative scenarios concern the hypothetical consumers’ exposure to the element under investigation (7). Subsequently, components and associated health outcomes are identified and selected for inclusion in the RBA. Each component is assessed individually, and when possible, its impact is converted into a common metric to facilitate comparison of scenarios.

A key step is to identify and prioritize the components and their associated health outcomes that should be considered in the RBA. This step is based on a literature search and, ideally, on a systematic review, considering the degree of evidence and the quality of data (1). To date, the identification and selection of components and associated health outcomes in RBA were performed mainly using non-harmonized and unstandardized experts’ judgment, lacking a harmonized strategy across the three areas of nutrition, microbiology, and toxicology. A recent systematic review containing 106 RBAs on fish and seafood has clearly highlighted how diverse this selection of components can be, even when investigating the same or similar foods (10). Nevertheless, the decision to include or exclude a component can possibly influence the results and conclusions of an RBA. Thus, this particular step, that is, “3.1 Nutrient(s) and hazard(s) identification” (Figure 1), needs to be strengthened by reporting components and linked health outcomes identified, by developing a method to rank/prioritize them and by making a justified and harmonized selection within the fields investigated.

In the area of microbiology, the prioritization and selection of significant microbiological hazards are well established by applying the principles used to establish the Hazard Analysis Critical Control Point (HACCP) system in which the first principle is to perform a hazard analysis. This includes listing all potential hazards associated with each manufacturing step considering the probability of a hazard to occur in view of possible contamination, survival, or proliferation of microorganisms in the food item and the severity of its health consequences (11). Thus, in this field, risk ranking strategies of biological hazards are well established and applied (12, 13). Such strategies were recently followed and extended by the French Agency for Food, Environmental and Occupational Health & Safety (ANSES) to rank foods associated with biological and chemical hazards, using a harmonized approach (14) based on multi-criteria decision analysis (MCDA) methods and defining specific criteria for each field. These methods were considered as the basis in the field of microbiology for the present work.

This study aims to establish a harmonized and transparent methodological framework for selecting the components from the areas of nutrition, microbiology, and toxicology to be considered in an RBA. The component selection is one of the first steps of an RBA preliminary to the model development.

The methodological framework was developed in a case study performed in the Novel foods as red meat replacers—an insight using Risk-Benefit Assessment methods (NovRBA) project (15). The case study aimed to estimate the overall health impact of replacing a red meat product with *Acheta domesticus* powder. Insects were considered in powder form in this substitution to increase their acceptance in Western countries (16).

## Materials and methods

To establish a harmonized strategy of selection and to attribute equal importance to each field (nutrition, microbiology, and toxicology), a three-step tiered approach was applied in all fields taking into account the specificities of each field upon the implementation of each step. This approach was developed considering existing practices in risk ranking (12), biological risk assessment (14), and HACCP system (17) and was further adapted to include nutritional and toxicological components.

**FIGURE 2:**
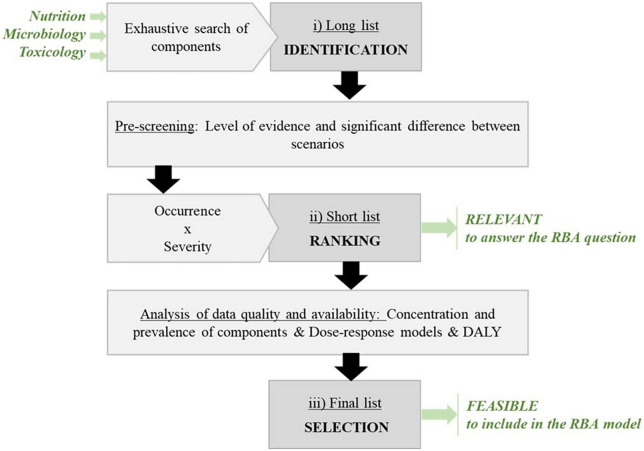
Scheme of key steps applied to establish a “long”, “short”, and “final” list of components.

The proposed framework established three lists of components: “long,” “short,” and “final” (Figure 2). The “long list” of components was established in each domain (nutrition, microbiology, and toxicology) based on a comprehensive literature search and included all the potential components to be considered in the RBA. This list was refined, and its elements were ranked to select top priorities to be included in the short list, considering specific criteria for occurrence and severity in each domain. The “short list” included all components that should be assessed to answer the posed risk–benefit question. The components included in the “short list” were further reviewed considering data quality and availability, toward the creation of the “final list.” The “final list” included all components considered for the RBA model. It should be noted that the “final list” is usually the unique list of components communicated in RBA studies, while the short list is extremely important because it includes all the component that have been identified as important to be evaluated with respect to the level of evidence. Components missing from the final list are therefore essential, and the inability to include them in the RBA should be communicated alongside the RBA results.

### Stepwise approach of component selection

**FIGURE 3:**
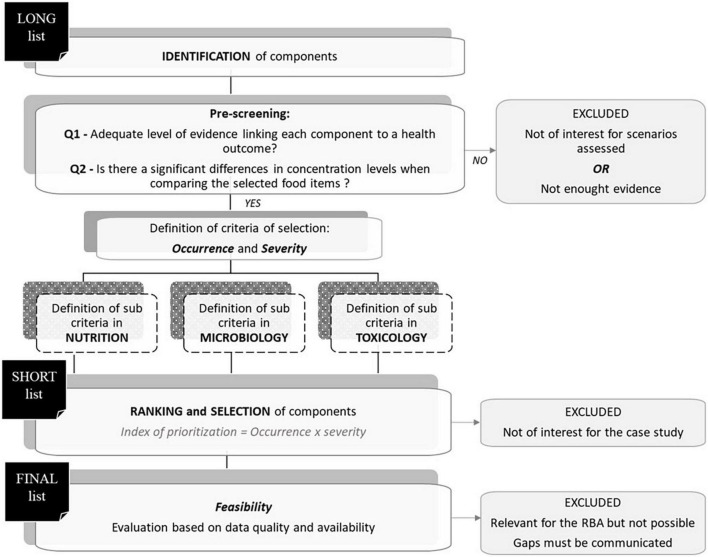
Flowchart of the standardized process to select food components (nutrients, microbiological, and chemical hazards) to be consider in the RBA.

The details of the component selection process are presented in Figure 3. In brief, the components for inclusion in the long list were initially identified *via* an exhaustive literature search. The resulting lists were subsequently reviewed to investigate the adequate level of evidence linking each component to a health outcome (question 1) and the differences in concentration levels when comparing the selected food items (question 2). The level of evidence was considered adequate when classified as “convincing”; components for which evidence of association with health outcomes was classified as “limited” or “contradictory” were excluded. If the reply to at least one of the questions—1 or 2—was “no,” the component was excluded. Subsequently, a set of standardized criteria were defined and applied in each domain considering data on the occurrence of the component under investigation and the severity of the associated outcome, assessed through a set of sub-criteria specific to nutrition, microbiology, and toxicology. The three resulting rankings were examined and discussed to set similar thresholds of selection in order to compile the short list. Hence, the short list finally included all the component–health outcome pairs that were identified as important to be assessed, given the level of evidence.

At the end, the final list included the components and health outcomes to be considered in the RBA to estimate the overall public health impact using the DALY composite metric. Excluded components that were considered relevant to answer the risk–benefit question but could not be included in the assessment due to data gaps must also be communicated to risk managers.

### Strategy of component identification to define the “long list”

The long list of components was assembled based on the literature search on each food item. Available data on nutrients and nutrient-related compounds, microbiological agents, and elements of toxicological concern were collected from the EFSA databases, national food composition databases, scientific publications, and repositories of national food safety agencies of the countries included in the assessment. In the area of nutrition, components quantitatively analyzed in one foodstuff but not in the other were included as it is about a substitution assessment. Food components present in both foods were included in the long list only if the difference between the two concentration levels was, in absolute terms, higher than 20%. The cutoff of 20% difference was defined on the basis of an acceptable 10% deviation due to analytical errors (method of analysis, sampling, etc.) and a minimum significant 10% difference when various foodstuffs are compared. Regarding toxicological and microbiological hazards, all hazards that were found in each food item were taken into consideration.

### Definition of sub-criteria of occurrence and severity in nutrition, microbiology, and toxicology to develop the “short list”

#### General ranking calculation (common in the three fields)

The ranking of components identified in the short list was based on a set of standardized criteria taking into account data on the occurrence (criterion 1) and the impact of the associated health outcome(s) (criterion 2) with specific definitions applied in nutrition, microbiology, and toxicology. Each nutrient, microbiological, and chemical hazard received a grade estimated by the product of the score achieved in each criterion. Hence,

INDEX⁢of⁢prioritization=Score⁢in⁢Criterion⁢1 ×Score⁢in⁢Criterion⁢2

Criteria 1 and 2 were first described in each domain, considering specificities of each field. Each criterion was defined creating 1–3 sub-criteria, and each sub-criterion had three levels attributed. Then, each component was examined and was attributed a corresponding level for each sub-criterion.

#### Definition of criteria in nutrition

**FIGURE 4:**
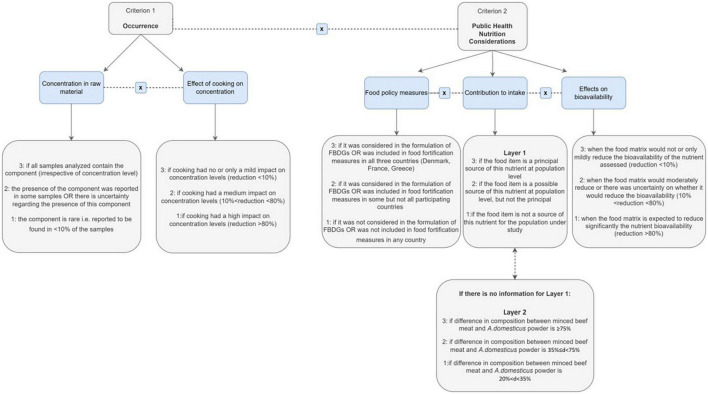
Definition of sub-criteria of occurrence and severity considered in nutrition.

For the selection of nutrients and nutrient-related compounds to be prioritized for inclusion in the short list, a standardized grading system was developed, pilot-tested, and finally applied based on the following two criteria: occurrence (criterion 1) and public health–nutrition considerations (criterion 2). Definitions and scores in each criterion and associated sub-criteria are presented in Figure 4.

In brief, occurrence was defined taking into consideration the concentration of each nutrient in the raw material together with the effect of processing on concentration levels (Figure 4). Therefore, a food component (e.g., protein or vitamin) was graded based to 3-point scales. In the first scale, scores ranged between 1 and 3, when the component was absent or not frequent, that is, reported in <10% of the samples (score 1) or was present in all samples analyzed (score 3). In case of uncertainty regarding the presence of a nutrient in samples identified, due to sampling, farming practices, or data source, values from national repositories were considered more robust than analytical results reported for a single sample. Regarding the effect of processing on the concentration of a food component (e.g., protein or vitamin), a score between 1 and 3 was given depending on the impact of the processing method on the concentration levels (Figure 4). The selection of cutoffs to define a score of 1, 2, or 3 was based on expert judgment and aimed to capture extreme changes in occurrence levels.

The public health nutrition considerations (criterion 2) related to whether the components of interest are included in current food policy measures; the contribution of the food item under consideration to the intake of the component of interest; and how the food matrix could impact the bioavailability of the nutrient. At first, judgments relied on the importance of the nutrient in the formulation of national and/or regional food-based dietary guidelines (FBDGs). In addition, the implementation of national food fortification schemes (e.g., foods fortified with this nutrient as a measure to promote public health) was also taken into account. A nutrient received a score between 1 and 3 if it was considered in the FBDGs or in food fortification measures in any or all countries under study, respectively (Figure 4).

The second sub-criterion considered the contribution of the foodstuff to the intake of each identified nutrient in the population under study (layer 1). When there was no information on the average contribution of the food to the intake of the nutrient, differences in the composition of the two food items regarding this nutrient were considered (layer 2).

Taking into consideration the impact of the food matrix on the nutrient bioavailability, a nutrient received a score between 1 and 3 based on the level of reduction (mild to significant) of the bioavailability of the nutrient assessed.

#### Definition of criteria in microbiology

**FIGURE 5:**
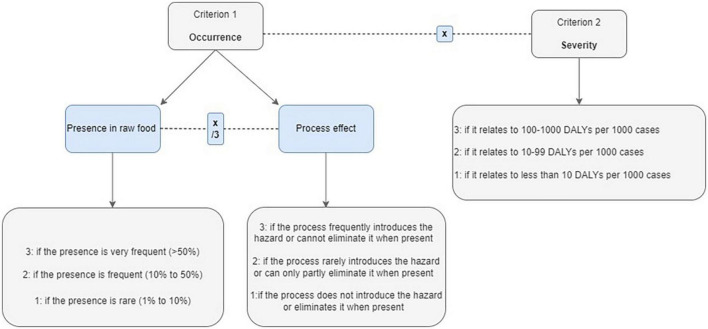
Definition of sub-criteria of occurrence and severity considered in microbiology.

In an approach similar to the one applied in the case of nutrients, the presence of microbiological hazard in raw food and processing effect were considered to estimate the occurrence of microbiological hazards (criterion 1). The impact of associated health outcomes expressed in DALYs was considered for severity (criterion 2). Criteria 1 and 2 were equally weighted. The score in criterion 1 derived through the multiplication of the two sub-scores in the presence of the microbiological hazard in raw food (prevalence) and the effect of the manufacturing process. This later received a score between 1 and 3 based on whether the process can reduce/eliminate the hazard or introduce it. The respective sub-criteria are defined in Figure 5.

Criterion 2 considered the severity of health outcomes associated with the exposure to microbiological hazards. For each hazard, severity was evaluated based on the mean DALY per case of disease with three ranges expressed as follows: <10, 10–99, 100–1,000 DALYs per 1,000 cases. The mean DALY encompasses the probability of getting different disease outcomes and the resulting quality of life lost, the associated durations, potential sequela, and years of life lost in case of premature death (18).

Finally, for the estimation of the overall grade in the prioritization index and the decision on whether a microbiological agent will be forwarded to the short list, the result of score of criterion 1 was multiplied by criterion 2 so that criteria 1 and 2 contribute equally.

#### Definition of criteria in toxicology

**FIGURE 6:**
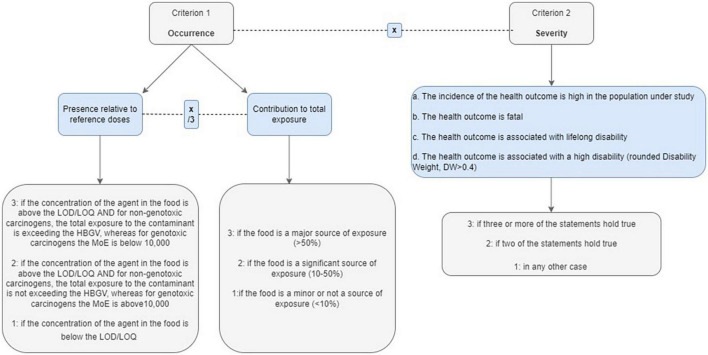
Definition of sub-criteria of occurrence and severity considered in toxicology.

For selection of chemical hazards, criterion 1 included two sub-criteria: “the presence relative to reference doses” and “the contribution to total exposure” (occurrence), and criterion 2 considered the “impact of associated health outcomes” (severity). The respective sub-criteria are defined in Figure 6.

With respect to criterion 1 on “occurrence,” the sub-criterion on “contribution to total exposure” was defined considering the concentration of the hazard in the food if it was above the limit of detection/limit of quantitation (LOD or LOQ) and whether the total exposure to the contaminant was exceeding the health-based guidance value (non-genotoxic carcinogens) or the margin of exposure (MoE) was below 10,000 (genotoxic carcinogens). The score was 3 when these two points were found, 2 if the total exposure to the contaminant was not exceeding the HBGV, whereas for genotoxic carcinogens, the MoE was above 10,000, and 1 when the concentration of the agent in the food is below LOD or LOQ. The sub-criteria on “contribution to total exposure” considered the proportion of exposure from the food assessed in the total diet with three levels: >50, 10–50, and <10%.

Criterion 2 considered the severity of the health outcomes associated with the exposure to the chemical hazard. The severity was evaluated qualitatively and considered the input parameters for the DALYs. The score (1 to 3) was based on how many of the following statements hold true:

(a) The incidence of the associated disease/condition is high in the population under study.
(b) The disease is fatal.
(c) The disease is associated with lifelong disability.
(d) The disease is associated with high disability (rounded disability weight (DW) > 0.4).

At the end, criteria 1 and 2 were multiplied, contributing equally to the overall grade of a chemical hazard.

Finally, the obtained rankings were analyzed to set a limit of inclusion in the short list. The same limit was applied for hazards in microbiology and toxicology for both food items, attributing the same importance to each component and harmonizing the selection. In nutrition, where a large list of components was established, the index of prioritization was calculated with a larger scale to enable a detailed ranking. Thus, the limit set was different but enabled a more objective selection between all components and the two foods.

### Check list of data required for each component to define the “final list”

The final list comprised components to be included in the RBA that aimed to quantify the health impact into the DALY composite metric. For each component, certain data were necessary to ensure the RBA model implementation and DALY calculation. This list is specific to the kind of assessment performed for each component, taking into account the particular characteristics of the fields of nutrition, microbiology, and toxicology. To enable a DALY calculation, it is necessary to estimate the potential increase or decrease in the number of cases for each health outcome. The main inputs required for each component are listed in Table 1. For nutrients and hazards characterization, the availability of dose-response data and incidence of health outcomes, as well as source attribution data (specifically for microbiological components) are required. The exposure assessment is based on current and alternative food intake data of selected scenarios as well as the availability of values on food composition and contamination (including prevalence and concentration). Composition and concentration could be collected directly with regard to the food item or considering the process effect. Finally, the DALY calculation requires inputs on the DALY value per health outcome case. In the absence of any of these data, the component will not be included in the final list for the quantitative estimate but must be considered a source of uncertainty in the health impact obtained and a priority for next improvement.

**TABLE 1:** List of data required for feasibility of the NovRBA case study.

RBA step concerned		Inputs required
Nutrient(s) and hazard(s) characterization *(Step 3.2*, Figure 2)	Dose-response	° Dose-response data for each health outcome ° Source attribution data (when applicable, e.g., in microbiology) ° Incidence of each health outcome (national data)
Exposure assessment *(Step 3.3*, Figure 2)	Food intake	° Current food intake (national data with variability) ° Alternative food intake calculation
	Composition	° Nutrient concentration in food items selected (national data with variability) ° Effect of processing on nutrients
	Contamination	° Chemical hazards’ prevalence and concentration (national data when available) ° Microbiological hazards’ prevalence and concentration(national data when available) ° Conditions of temperature and storage (duration and temperature) all along the farm to fork chain ° Models of inactivation and growth of each microbiological hazard
Risks and benefits characterization *(Step 3.4*, Figure 2)	DALY calculation	° DALY/case for each health outcome

## Results

### “Long list” of components identified in beef and crickets

The long list of components is presented in Table 2. It was completed based on the systematic review approach for *A. domesticus* (19). The recently published EFSA opinion on the safety of frozen and dried formulations from *A. domesticus* as a novel food (20) was also considered. For minced beef, profiles were based on key information sources in each domain, including EFSA databases and national food composition tables. Data on the composition of minced beef meat were retrieved from the Danish and French Food Composition Tables (21, 22), due to the unavailability of such data in the Greek food composition database.

**TABLE 2:** The “long” list of components identified in beef meat and crickets (selection made through the NovRBA case study).

Nutrients			Microbiological hazards		Chemical hazards	

Crickets and beef			Crickets	Beef	Crickets	Beef
**Macronutrients:** Carbohydrates Fiber Fiber IDF (insoluble) Fiber SDF (soluble) **Minerals:** Aluminum Calcium Chloride Chromium Copper Iodine Iron Magnesium Manganese Potassium Selenium Sodium Sulfur Zinc	**Vitamins:** Folate Niacin Pantothenic acid Retinol Thiamin Cyanocobalamin Riboflavin Pyridoxine Vitamin C Vitamin D3 (Cholecalciferol) Vitamin E (alpha-Tocopherol) **Fatty acids:** Total MUFAs Total PUFAs Total n-3 fatty acids Total n-6 fatty acids Total SFA	**Sterols:** Cholesterol **Amino acids:** Alanine Cystine/cysteine Histidine* Lysine* Methionine* Phenylalanine* Tryptophan*	**Bacteria**: *B. Cereus* *Campylobacter spp.* *C. Botulinum* *C. perfringens* *Cronobacter sakazakii* *EHEC* *Listeria monocytogenes* *Salmonella* *S. aureus* *Vibrio sp.* *Yersinia enterocolitica* **Virus:** *HVA* *Norovirus* **Metabolites:** *Histamine*	**Bacteria**: *B. Cereus* *Campylobacter spp.* *C. perfringens* *Cronobacter sakazakii* *EHEC* *Listeria monocytogenes* *Salmonella* *S. aureus (rare, human origin)* *Yersinia enterocolitica* **Virus:** *Hepatitis E* *Norovirus (human origin)* *Rotavirus (human origin)* **Parasites:** Cryptosporidium spp. (veals) Toxoplasma gondii	**POPs:** Total PCB (organochlorine compounds) Phosphorous flame retardants (PFR, organochlorine compounds) Oxychlordane (OCD, flame retardant) Polybrominated diphenyl ethers (PBDE, flame retardant) **Metals:** Mercury Cadmium Lead Inorganic arsenic Aluminum Nickel Chromium **Pesticides:** (presence of 10 pesticides)	**POPs**: Total PCB Dioxin + dl-PCBs Organochlorine compounds (chlordane, hexachlorobenzene, dieldrin, lindane, DDT) **Metals:** (Methyl)mercury Cadmium Lead Inorganic arsenic Aluminum Nickel Chromium **Pesticides:** [organochlorine compounds listed above] **Neoformed compounds or** **process contaminants:** PAH

The long list for minced beef and cricket powder included 42 and 41 nutrients and nutrient-related components, 13 and 14 microbiological hazards, and 10 and 12 chemical hazards, respectively.

### “Short list” of components

The ranking of components was based on scores attributed to each sub-criterion presented in section “Definition of criteria in nutrition.” All values attributed to components in minced beef and cricket powder are provided for illustration purpose in the Supplementary material. For each nutrient, microbiological, and toxicological components, an index of prioritization was estimated by combining the score of occurrence and severity. Each criterion was calculated based on one, two, or three sub-criteria. In microbiology and toxicology, which are both related to food safety hazards, the same weight was attributed to occurrence and severity, giving an index of prioritization varying between 1 and 9. The limit of inclusion in the short list was set at 2 in both domains considering a main public health concern in both domains. In nutrition, the nature of compounds represents a major difference from the hazards because they constitute each food and they are naturally present. The index of prioritization was also based on the multiplication of criterion of occurrence and severity with two and three sub-criteria applied, respectively. This allowed for a broader scale, necessary to rank 43 nutrients, with an index of prioritization ranging between 1 and 243 points. The threshold applied was set at 108 for both food items, enabling an equal consideration.

The short list of components regarding minced beef and cricket powder is described in Table 3. It included 9/44 and 10/44 nutrients, 5/13 and 6/14 microbiological hazards, and 2/11 and 1/12 chemical hazards (values representing the number of compounds in short/long lists) for minced beef and cricket powder, respectively.

**TABLE 3:** The “short” list of components identified in minced beef and cricket powder (selection made through the NovRBA case study).

Nutrients		Microbiological hazards		Chemical hazards	
AD	Beef	AD	Beef	AD	Beef
Calcium Copper Fiber (including chitin) Iron Magnesium Selenium Sodium Total n-3 fatty acids Total n-6 fatty acids Zinc	Iron Niacin, Selenium Sodium Total saturated fatty acids Thiamin Cyanocobalamin Vitamin D3 Zinc	*Bacillus Cereus* *Clostridium Botulinum* *Clostridium perfringens* *Cronobacter sakazakii* *Listeria monocytogenes* *Salmonella* spp. *S. aureus* (enterotoxin)	*Clostridium perfringens* *Campylobacter* spp. *Listeria monocytogenes* *Salmonella* spp. *S. aureus* (enterotoxin) *Toxoplasma gondii*	Inorganic arsenic	PAH

### “Final list” of components

The final list (Table 4) corresponds to components that were included in the short list, feasible to be assessed quantitatively and finally of relevance to be integrated in the RBA model. (Details on the step-wise approach applied are given in Table 1.) In nutrition, nutrients that were included in the short list of only one food were systematically considered for the second food to assess the change in nutrient exposure when making the food substitution. This does not apply to microbiology and toxicology, where the presence of a hazard corresponds to a contamination. In these fields, each food was considered independently. At the second step, selection relied on the availability of data on dose–response associations and on DALYs associated with health outcomes. Dose–response associations between macro- and micro-nutrients and health outcomes have been identified through a PubMed Search, followed by a citation checking of key papers retrieved. Focus was given on hard endpoints (i.e., incidence of disease), excluding data on intermediate factors (e.g., blood pressure and markers of glucose metabolism or inflammation). Estimates for DALYs/case were based on values reported, or after the division of total DALYs per incidence rates for the selected diseases, extracted from the Global Burden of Disease (GBD) database and European sources for microbiological hazards (23).

**TABLE 4:** The “final” list of components identified in beef meat and crickets (selection made through the NovRBA case study).

Nutrients	Microbiological hazards		Chemical hazards	
		
AD and beef	AD	Beef	AD	Beef
Calcium Fiber, insoluble fiber Iron Magnesium Sodium Vitamin B12 Zinc	*Bacillus Cereus* *Clostridium perfringens* *Cronobacter sakazakii* *Listeria monocytogenes* *Salmonella* spp.	*Clostridium perfringens* *Salmonella* spp. *Toxoplasma gondii*	/	Inorganic arsenic

Reasons of exclusion from the short to the final lists are collectively listed in the Supplementary material. The final list of components of minced beef and cricket powder is provided in Table 4. It included 7/9/44 and 7/10/44 nutrients, 3/5/13 and 5/6/14 microbial hazards, and 1/2/11 and 0/1/12 chemical hazards (values representing the number of compounds in the final/short/long lists) for minced beef and cricket powder, respectively.

### Identification of associated health outcomes

**FIGURE 7:**
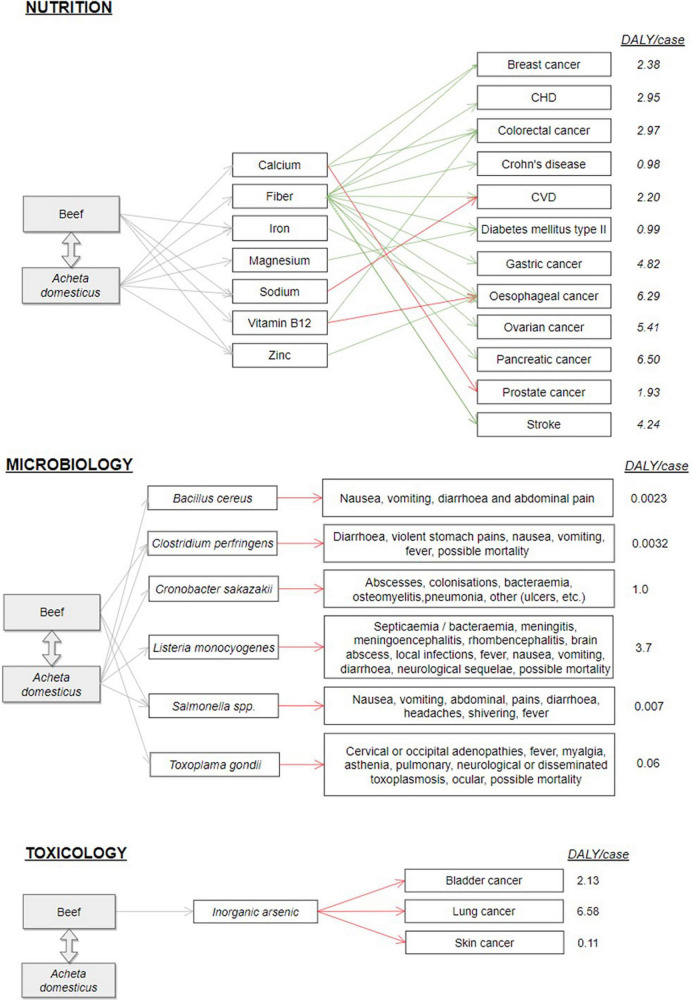
Tree of components of the final list and associated health outcomes identified in the NovRBA case study (red when the expected effect is negative and green when it is beneficial).

For each component included in the final list, associated health outcomes were identified (presented in Figure 7). In nutrition, EFSA Scientific Opinions on Dietary Reference Values were screened, and PubMed search focusing on systematic reviews and dose–response meta-analyses for hard endpoints (a scoping review of the literature, giving priority to epidemiological studies) was conducted to identify evidence on causal associations between exposure to the compounds of toxicological concern and adverse health outcomes in humans. Health outcomes related to microbiological hazards were collected from the literature, considering also epidemiological reports.

Figure 7 highlights the complexity of estimating the direction of the total health impact that can be overall positive or negative. Indeed, in microbiology and toxicology, only adverse health outcomes were studied, but the food substitution introduced, at the same time, a reduction of risks associated with minced beef by an increase in risks associated with cricket powder, and it may refer to the same hazard. Nutrients are often present in both foods at different levels, which are associated with a reduction or increase in risks. Furthermore, the same nutrient can induce both adverse and beneficial effects, depending, for example, on the intake levels. In case there is no clear outweigh of beneficial or adverse effect, RBA should rely on a common metric to estimate the overall health impact (7). Estimates for DALYs/case were based on values reported or the division of total DALYs per incidence rates for the selected diseases extracted from the Global Burden of Disease (GBD) database (23) and European sources for microbiological hazards.

## Discussion

The identification, prioritization, and selection of components to be included in the RBA alongside linked health outcomes constitutes a major challenge. In past assessments, this selection relied mainly on unstandardized experts’ judgment, reporting a qualitative justification of their choices. This was clearly showcased in a recent publication by Thomsen et al. (10), who reported noteworthy differences regarding the selection of components among the 106 RBA studies on fish and seafood reviewed. The final outcome of an RBA relies on the combined health impact associated with components included in the assessment. Hence, the inclusion/exclusion of one or several components of possible relevance might alter the outcome. Therefore, in the context of the NovRBA case study, we developed a strategy to select the RBA components related to nutrition, microbiology, and toxicology in a transparent, reproducible, and harmonized manner, by establishing specific steps and standardized criteria for the selection of the RBA components. This newly developed methodology was tested on the NovRBA case study, that is, the substitution of a widely consumed food (minced beef) by a novel food ingredient (cricket powder), which further highlighted challenges related to the food sources and amount of data available.

A three-step tiered approach was designed and tested within the NovRBA case study. The first step included the identification of a “long” list of components for minced beef and cricket powder, through a comprehensive literature search, accordingly. The “long list” comprised an exhaustive list of nutrients and nutrient-related components, microbiological agents, and compounds of toxicological concern for minced beef and cricket powder. The second step aimed to rank and select components of interest in each domain (nutrition, microbiology, and toxicology) by applying a harmonized strategy, toward the creation of the “short” list. In the last step, the “final” list included those components for which it was feasible from a technical and data availability point of view to be included in the RBA. The components included in the “short” list and the respective linked health outcomes, which were however, not prioritized for inclusion in the final list, must be communicated together with the results in order to highlight eventual data gaps and limitations, which are crucial elements to be considered in the decision-making process. The approach developed can be incorporated into the current methodological framework of RBA in the field of food and nutrition.

The NovRBA case study contained several assumptions that might have guided the suggested method development. First, the scope restricted the application to a quantitative health impact comparison of defined scenarios, using the disability-adjusted life years (DALYs) composite metric (18). A “food component-based approach” was chosen, meaning that only associations between health outcomes and specific food components were considered. The implementation of a boiling step in the manufacturing process of cricket powder was assumed as it is a classical and essential step to reduce the levels of microbiological hazards (24–27). Moreover, cricket powder produced by oven-drying was considered since this drying method is the most used currently in industry (28).

### Main advantages and limitations in applying the method developed

The strategy developed applies for assessments that are conducted at the component level and needs to be adapted in case of inclusion of associations between health outcomes and consumption of specific food. In the case study used in the current work, this later piece of information was rather limited for beef and absent for *A. domesticus*, a novel food.

The development of the prioritization index was based on severity and occurrence, which should contribute equally in order to give the same importance to nutritional, microbiological, and toxicological components. This facilitated a similar consideration of a dietary staple for which we have data over a long period of time versus a novel food with a large number of data gaps. Regarding the calculations applied in this analysis, some adaptations were considered in nutrition as the public health impact (severity) was considered more important than occurrence since nutrients are naturally present and available from a variety of foods compared with microorganisms and chemicals that are due to contamination. We therefore applied a proportional weight of sub-criteria selected (3/5 for severity and 2/5 for occurrence) in the field of nutrition. In the field of microbiology and toxicology, these two criteria had the same weight. This calculation was adapted from Naska et al. (15) in order to harmonize the calculation method between the three domains. This did not change the final list of components to be included in the RBA.

The long list of nutrients was also more comprehensive than that of contaminants, so all the sub-criteria were multiplied in nutrition, leading to a score ranging between 1 and 243, while the sub-criteria of severity and occurrence were equally weighted for contaminants, leading to a score ranging between 1 and 9. This enabled us to expand the nutrient ranking. The choice of a threshold to select components for the short list based on the long list remains a subjective decision of the assessors but is informed by objective quantitative elements that are fully transparent.

Finally, applying this method requires additional time and further research to justify each score attributed to sub-criteria, compared with classical selections based on experts’ judgment. On the other hand, this investment improves quality in the choices made, enables identification of gaps and future steps, and could lead to a more objective evidence-based estimation of the overall health impact.

### DALY metric and feasibility constraints

To date, in the food RBA field, three different strategies were used for scenario comparison (2) based on health-based guidance values (HBGVs), specific endpoints or DALY. The most common method is to compare the estimated levels of consumers’ exposure to a component with established HBGVs such as the tolerable weekly intake (TWI) in toxicology and dietary reference values (DRVs) in nutrition. This is the easiest way to proceed, with the main limitation being that it attributes the same importance to all potential health outcomes without considering the associated severity. The very wide variety of effects included in the present case justifies a different consideration. Moreover, the exceedance (or non-achievement) of an HBGV may not directly lead to a potential health outcome. The second method compares a change in a specific endpoint (e.g., the number of deaths). Both the methods would not be possible to be applied to the NovRBA case study due to the wide variety of health outcomes identified. Thus, the third method that includes the use of a composite metric such as DALYs was considered in the NovRBA case study, combining both the quality and quantity of life lost due to a disease. This was less frequently applied in the past because it requires a finalized and quantitative assessment of each risk and benefit, up to the estimate of the number of cases, something not always possible due to lack of dose–response data (10, 29). The DALY indicator might be attractive for managers to compare and rank several risk management options as it integrates the whole complexity of the RBA issue within a simple figure. However, the choice of the DALY metric has limited the inclusion of certain components in the model due to data gaps and thus reduced the list of components in the “long” list as it required dose–response data for all the “component–health outcome” and DALY values per case. This was illustrated in the present case with the inclusion of 16 components in the final list compared with 28 included in the short list. Consequently, the selection of the DALY metric will thus also influence the main conclusions of an RBA study. The choice of the most efficient way to compare health impacts in RBA belongs with the general debate on the measurement of health (30) as it is an indirect measurement of a selection of indicators representing “the conception of health” (31). In RBA, there is no consensus on a metric to compare scenarios; it needs to be defined by taking into account each RBA context and anticipating potential public health-related measures for the implementation of a fit-for-purpose assessment.

### The weight of evidence of “component(s)–health outcome(s)”

Risk–benefit assessment involves various health outcomes for which the current scientific weight of evidence associated with their exposure to the food items of interest may vary (32). This weight of evidence reflects the degree of current scientific knowledge regarding the association between the consumption of each component and the occurrence of a certain health outcome, that is, the “biological knowledge of the day” (33). For instance, health outcomes for which the biological mechanism has been proved in humans are associated with a stronger level of evidence than others for which a relationship has been only suspected in animals or in *in vitro* studies. The level of evidence might also differ between nutritional, microbiological, and chemical hazards. This fact was considered in the selection of health outcomes by using only dose–response studies with a convincing level of evidence, but could be more clearly included as suggested in BRAFO tables (34) with a narrative description. It could also be addressed quantitatively by considering a “probability of causation” based on experts’ elicitations, as proposed by Trasande et al. (35).

### From risk–benefit assessment to a multiple risk assessment

Risk–benefit assessment aims to estimate the overall impact of food consumed on consumers’ health for different scenarios of dietary exposure. Implicitly, RBA serves to highlight possible ways of improving public health through specific dietary choices. However, “health” is a concept with several dimensions, which cannot be measured like a biological parameter such as weight or length (30). Health was defined as “a state of complete physical, mental and social well-being and not merely the absence of disease or infirmity” (36). In that respect, considering “benefits” in RBA might refer to improving health, corresponding to an increase in the “level of functioning or capacity in all the important dimensions of health, and from any type of illness or disease” (37), as well as to reduce the risk of premature death. Nevertheless, as illustrated in the NovRBA case study and also in previous RBA studies (29), health outcomes included in RBA are related to the increase or prevention of “adverse health outcomes” associated with illness and/or premature death, instead of “beneficial health outcomes” that could be seen as an increase of the health status toward a high level of wellness. Consequently, RBA corresponds in a semantic way rather to a multiple risk assessment. Thus, classical methods of risk ranking (12) and MCDA (38) might be of interest to develop the RBA methodology further.

### Future perspectives

An additional public health-related challenge that emerged within the NovRBA case study relates to potential allergenicity aspects linked to the consumption of cricket powder. Allergenicity assessment of novel proteins and their sources remains a complex area, subject to further improvement and standardization (39, 40). Moreover, considering the one-health approach (41), other interconnected factors will influence the decision-making process. Thus, RBA should not be seen as a one-dimensional process but as a process that could benefit from the additional integration of environmental, economic, and sociological assessments to underpin, for example, sustainable foods and diets.

## Data availability statement

The original contributions presented in this study are included in the article/Supplementary material, further inquiries can be directed to the corresponding author.

## Author contributions

GB, EV, and ANa: conceptualization. GB, EV, ANi, and ANa: literature search, title/abstract screening, full-text screening, and writing – original draft. GB, EV, ANi, ST, and ANa: data extraction and data curation and visualization. GB, EV, ANi, MF, SP, MP, ST, and ANa: writing – review and editing. All authors contributed to the methodology and data interpretation.

## Author disclaimer

EV is employed with the European Food Safety Authority (EFSA) in the Nutrition and Food Innovation Unit that provides scientific and administrative support to the panel on nutrition, novel foods, and food allergens, in the area of novel foods. However, the present article is published under the sole responsibility of the authors and may not be considered an EFSA scientific output. The positions and opinions presented in this article are those of the authors alone and do not necessarily represent the views or scientific work of EFSA. To learn about the views or scientific outputs of EFSA, consult its website at http://www.efsa.europa.eu that aided the efforts of the authors.
